# How the vagus nerve produces beat-to-beat heart rate variability; experiments in rabbits to mimic in vivo vagal patterns

**Published:** 2015-12-20

**Authors:** John M. Karemaker

**Affiliations:** Department of Anatomy, Embryology and Physiology, Academic Medical Center, University of Amsterdam, Amsterdam, the Netherlands

**Keywords:** sinoatrial node, vagotomy, phase response curve, respiratory sinus arrhythmia, baroreflex, cardiac autonomic plexus, cardiac baroreflex

## Abstract

**Background and Aim:**

Analysis of heart rate variability (HRV) has recently become the playing field of mathematicians and physicists, losing its relation to physiology and the clinic. To set the record straight, a set of animal experiments is presented here, which was designed to test how vagus nerve traffic might produce beat to beat (b-t-b) heart rate (HR) control, like the baroreflex will do in vivo.

**Methods:**

The response of HR to vagus nerve stimulation was tested after bilateral vagotomy in rabbits under anesthesia. Three protocols were followed: 1. Single burst stimulation at varying moments in one cardiac cycle; 2. B-t-b stimulation in each cycle, coupled to the P-wave with variable delays; in addition, testing the effects of one increased or decreased burst; 3. Tetanic stimulation, shortly interrupted or increased at varying moments in the cardiac cycle.

**Results and Conclusions:**

Sensitivity of the sinoatrial node to the timing of vagal bursts in its cycle from protocol 1 explains most of the observations. A single burst would be most effective when applied in late repolarization or early diastole of the sinoatrial node’s action potential. In b-t-b stimulation the longest cardiac cycles occur when bursts are timed just before the end of the ‘sensitive period’. Later coming bursts have their (diminished) effect on the next cycle; critically timed bursts induce an unstable HR, alternating between long and short cycles. This ran in synchrony with the respirator, thus producing a large respiratory sinus arrhythmia, even though the vagus nerves had been cut. HR-response to vagal burst activity shows two components: a fast one which is phase-sensitive and a slow one that builds up with longer lasting activity and also disappears slowly. Tetanic stimulation results in prolonged, but variable cycle lengths which are difficult to change by short-lasting manipulation of impulse frequency, be it up or down.

**Relevance for patients:**

Measurement of heart rate variability (HRV) and baroreflex sensitivity (BRS) have become clinical tools in the cardiology clinic and in hypertension research. This study shows how the underlying vagus nerve to heart rate physiology is responsible for moment-to-moment variability in these numbers at almost unchanged underlying physiology. Programmed stimulation of the vagus nerves in acute animals (rabbits) demonstrates that the optimal mode of fast, beat-to-beat heart rate control by these nerves is by means of bursts of impulses arriving in every heart beat at well-timed moments. In vivo this is how the baroreflex stabilizes blood pressure at the expense of HRV.

## 1. Introduction

**Figure 1.**
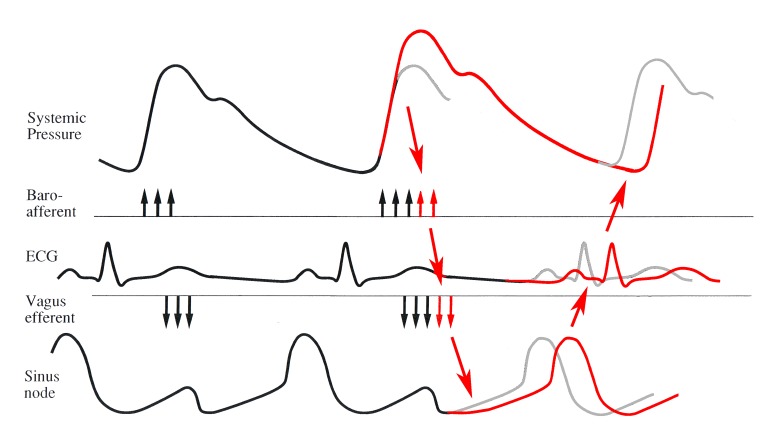
Blood pressure control by the fast (vagally mediated) baroreflex. Top trace blood pressure, next schematic baroreceptors afferent signal, (ECG for time reference), then cardiac vagus nerve efferent activity, delaying the sinus node’s depolarization (lower trace). In the second beat systolic blood pressure is increased (red line) by an increased stroke volume; the baroreceptors react by more afferent impulses, resulting in more efferent vagal activity, which is delaying the upcoming beat by longer hyperpolarizing the sinus node. Consequently, the next diastolic pressure is not as much increased as the grey lines suggests but already more or less stabilized. Figure adapted from [2] and [3].

Heart rate variability is, boldly put, the price paid by the blood pressure control system to obtain blood pressure stability. Figure 1 demonstrates this hypothesis schematically: the blood pressure upstroke induces baroreceptor afferent impulses at each heartbeat, which are immediately turned into vagus nerve efferent activity to slow down the sinoatrial node. If, for instance, the heart produces a larger stroke volume (the second beat in the schema) this is sensed by the baroreceptors and transformed via the vagus nerve into more slowing down. Consequently, the next diastolic pressure is already more or less stabilized, depending on the effectiveness of the baroreflex. This idea on how the vagus nerve helps control blood pressure is the center piece of the ‘DeBoer’ model [1] which helps to understand the relationship between heart rate and blood pressure variability in daily life.

Heart rate variability (HRV) is also a reflection of the autonomic nervous system: sympathetic and parasympathetic (vagus nerve) activity, and circulating hormones. The fast beat-to-beat changes can be attributed to changes in vagal activity. This makes HRV the ideal candidate for non-invasive and almost continuous observation of a person’s autonomic condition: little HRV and higher heart rates would imply sympathetic (over-)activity, lower heart rates with large beat-to-beat changes imply tranquility with parasympathetic domination. This maxim has ruled the field of HRV-analysis for a long period of time. Recently it was challenged from 3 angles: 1) the observation that over a long range of heart rates in different species HR and HRV are strongly related, leading to the notion that HRV is just another way of looking at HR [4,5], combined with: 2) New analysis of sinoatrial node physiology, stressing that the pacemaking process itself is subject to inherent variability, due to the properties of the tissue and the surrounding micro-milieu [6]. 3) The heart has integrating nervous centers of its own in the cardiac ganglionic plexuses, where integration of autonomic influences and instantaneous cardiac demands take place [7]. In the present paper a synthesis of the old and new views is attempted, based on vagus nerve-sinoatrial node physiology in vivo. For this purpose, an old set of animal experiments has been re-analyzed. Earlier, the results had only been presented orally, focused on the oscillator properties of the sinoatrial node [8]. Recently a partial result of the re-analysis has been presented as abstract [9].

Two modes of activity have been observed in efferent cardiac vagal traffic [10-12]: in pulsatile mode there are bursts of impulses in each cardiac cycle, reflexly coupled to the upstroke of the pulse wave, sensed by baroreceptive afferent areas in the circulation; in tetanic mode, vagal activity is more or less continuous, loosely coupled to the level of blood pressure, but also to chemoreceptor- and other afferent nerve traffic. In the present experimental animal study both modes of efferent cardiac vagal activity have been tested for their aptitude to induce changes in heart rate (HR) from one beat to the next.

In the literature a large number of papers can be found on heart rate responses to vagal stimulation, as reviewed in [13]. However, although short-lasting bursts of stimuli and repeated bursts have been tested for their ability to pace and synchronize heart rate, as in [14] no studies are available that looked at variations in burst-amplitude (i.e. number of applied stimuli per heart beat as mode of heart rate regulation.

**Figure 2.**
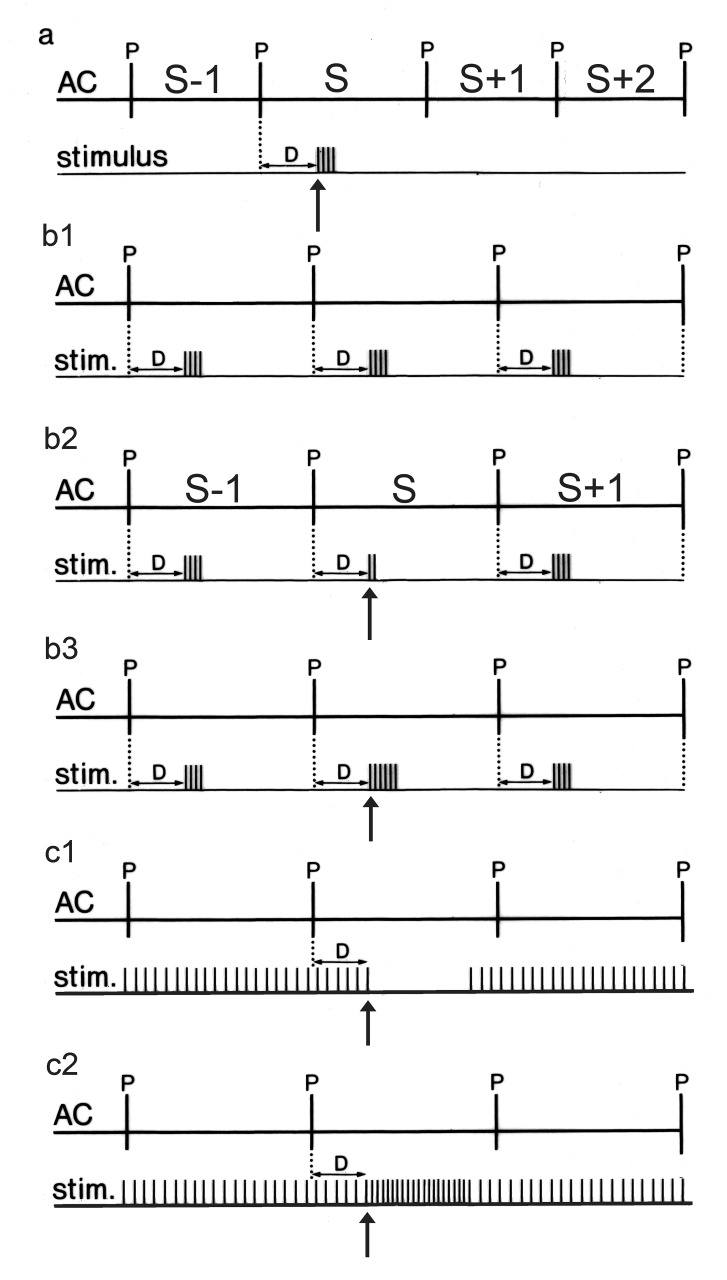
Schematic overview of the stimulation protocols. AC = atrial complex (indicating start of P-wave); D = variable delay between start of P-wave and stimulus or intervention. 2a. Single burst stimulation: burst in one cardiac cycle only at a variable delay D from start of the cycle. Number & strength of the pulses in the burst are adapted to the intended maximal amount of cycle lengthening. Responses of stimulated cycle (S) and later (S+1, S+2, …) are referenced to the last prestimulus cycle (S-1). 2b1. Beat-to-beat stimulation: one burst of stimuli at a variable delay D in each cardiac cycle. The delay is kept constant until a steady state is reached. 2b2. In one particular cycle (S) the strength of the stimulus burst is changed, either by decreasing (2b2) or increasing (2b3) the number of pulses in the burst. 2c1. Tetanic stimulation; in one particular cycle pulses are suppressed (2c1) or the frequency is increased (2c2) for an adjusted period, starting at a variable delay D from the beginning of the cycle.

**Figure 3.**
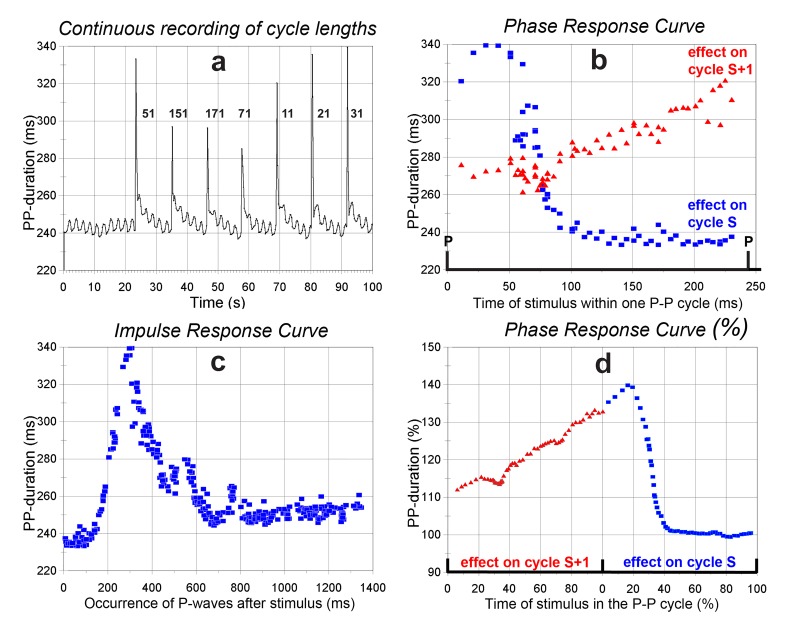
P-P cycle durations in response to a series of 61 stimulus burst applications in one cycle/12 seconds (burst: 4 pulses, 200 Hz, 0.3 ms duration, 9 V). 3a. P-P cycle recording of the first 100 seconds of the series; the numbers show the delay settings in ms for each stimulation. Note the small respiratory sinus arrhythmia. 3b. Phase response curve of the combined results. X-axis: delay D setting (Figure 2a). Y-axis, blue squares: resulting duration of the first (stimulated) cycle (S); red triangles: of the next (unstimulated) cycle (S+1). 3c. Impulse response curve. X-axis: time between the start of the burst and the occurrence of a P-wave for that run. 3d. Phase response curve (%); same data as in 3b, all data referenced to the last P-P cycle before the stimulus (in % of (S-1)-duration). Unstimulated second cycle (S+1) placed next to the stimulated one (S), to show the continuity of the phase response curve (data 5-points averaged).

When it comes to the question of how fast heart rate can react to the vagus, a classical approach is to systematically shift a strong burst of vagal activity through the cardiac cycle and measure its effect on the ongoing and following cycle durations (Figure 2a). Since the timing of the vagal burst does not only decide which cycle is influenced - the ongoing or the next - but also how much these are changed, the ensemble results of these experiments lead to so-called ‘phase response curves’ (PRC’s) [15-17] where the induced cycle prolongation is shown as function of the timing (phase) of the vagal burst in the ongoing cycle (an example of such a curve is found in Figure 3b). Such experiments have been done here as well. When considering the resulting curves, a number of questions came up:

1. Will the PRC change when heart rate is lowered by concurrent vagal stimulation, to mimic a changed vagal tone?

2. Is there a PRC for short-lasting decreases of vagal activity, i.e. for an increase in heart rate? Of course, this can only be measured when a constant level of background vagal activity is generated.

3. In relation to this: is there a difference in heart rate responses, either up or down, depending on how this background vagal activity is generated: pulsatile or tetanic?

The present set of experiments was designed to tackle the above-mentioned questions. Constant vagal restraint was induced either pulsatile (Figure 2b1) by a short burst of pulses in each cardiac cycle systematically shifting its timing in the cycle, or tetanic (Figures 2c1 and 2c2) by applying a constant frequency of stimulation to one vagus nerve. The sensitivity to changes in vagal activity and the inherent delays were tested by either increasing or decreasing the number of pulses in the bursts or by shortly increasing the frequency or stopping the tetanic stimulation and shifting that ‘block’ through the cardiac cycle. Alternatively, a short burst of pulses was applied to one vagus nerve while the other one was stimulated tetanically, so as to mimic the effect of ‘recruitment’. Figure 2 explains the various stimulation protocols.

These experiments had been designed to obtain qualitative rather than quantitative results: in one and the same experiment stimulus parameters were changed to observe differences in heart rate response in that animal. In view of the multitude of biological and experimental variables it is a rather futile attempt to quantify ‘the rabbit’s sinus node response’. Moreover, the experiments served to better understand human physiology in the end.

## 2. Materials and Methods

The experiments have been carried out over a period from 1979 to 1984, in accordance with prevailing law at the time and code of ethics in animal experimentation (Declaration of Helsinki, the UFAW handbook on the care and management of laboratory animals [18]), under supervision of the veterinary staff of the Jan Swammerdam Institute where the experiments have been conducted. Fourteen New Zealand White rabbits of both sexes have been used. Anesthesia was obtained and maintained by intravenous injection of Nembutal (Abbott, sodium pentobarbital, initial dose up to 30 mg/kg, followed by half the initial dose/hour as needed). In the last 9 experiments N2O/O2 was given as inspiratory gas after anesthesia induction in combination with the pentobarbital. This resulted in lower baseline heart rates than by pentobarbital alone. Analgesia to allow skin incisions was obtained by local infiltration with 2% lidocaine. At the end of the experiments the animals were euthanized by pentobarbital overdose.

A midline cervical incision was made and a tracheal cannu-la was inserted. Artificial ventilation was applied when necessary by use of Keuskamp’s ‘Amsterdam infant ventilator’ (Loosco, Amsterdam). Body temperature was maintained by a built-in heating in the animal table. After bilateral vagotomy at the mid-cervical level the peripheral end of the right vagus nerve (and/or the left nerve, if necessary) was placed in a snugly fitting flexible bipolar platinum electrode as described elsewhere [19]. ECG was recorded from silver electrodes placed under the skin of the thorax. A bipolar catheter mounted electrode (4F, diameter 1.35 mm) was introduced in the right internal jugular vein and positioned where it would detect a well-defined atrial complex. This atrial complex was used to trigger the timing circuits of the stimulation logic circuitry. Electrical stimulus pulses were produced by way of a 4710 dual channel Ortec stimulator (Ortec Inc., Oak Ridge, TN, USA), pulses were applied to each electrode by way of a Grass S5 stimulus isolation unit (Grass Instruments, Quincy, MA, USA). Pulse amplitudes and durations were limited to preferentially stimulate myelinated nerve fibers (max 10 V, 1.0 ms duration). Within these limits, effectiveness of stimulation was, as it were, titrated by varying the number of pulses within one burst. Changing either pulse duration or voltage would change the number of effectively stimulated nerve fibers, thereby complicating the comparison between responses. By increasing or decreasing the number of impulses within one burst would at least the vagal stimulation input to the sinoatrial node be changed linearly. This is of particular importance for the experiments described in figures 2b2 and 2b3 (beat-to-beat stimulation with once decreased or increased burst strength). This way of manipulating stimulation strength also helped to limit the amount of current passed by the stimulus electrode and to prevent damage to the nerve.

The P-wave trigger, stimulus-burst trigger and experiment status pulses were fed directly into a DEC PDP 11/40 computer (Digital Equipment Corp., Maynard, Mass. USA) fitted with a Lab Peripheral System, where the events were timed by a crystal clock, at a resolution of 0.1 ms. Stimulus-response curves were produced online. Trigger and status pulses, ECG and atrial complex were also FM-recorded by an Ampex FR 1300 (Ampex Corp, Nivelles, Belgium) on magnetic tape as backup. A Brush 481 polygraph pen recorder served for overview of heart rate responses.

Three stimulation protocols were followed (Figure 2).

### 2.1. Single burst stimulation

**Figure 4.**
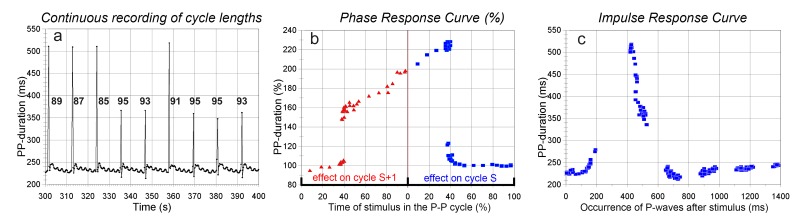
Cycle durations in response to a strong stimulus (8 pulses, 200 Hz, 0.5 ms duration, 10 V) to the left vagus nerve (layout as in Figure 3). 4a: excerpt from the cycle duration vs. time recording; numbers show the actual settings of the timing of the burst in the P-P cycle. A sharp transition between delays 91 and 93 ms is observable. 4b: Phase response curve (%) over 2 cycles from the same data. 4c: Impulse response representation.

**Figure 5.**
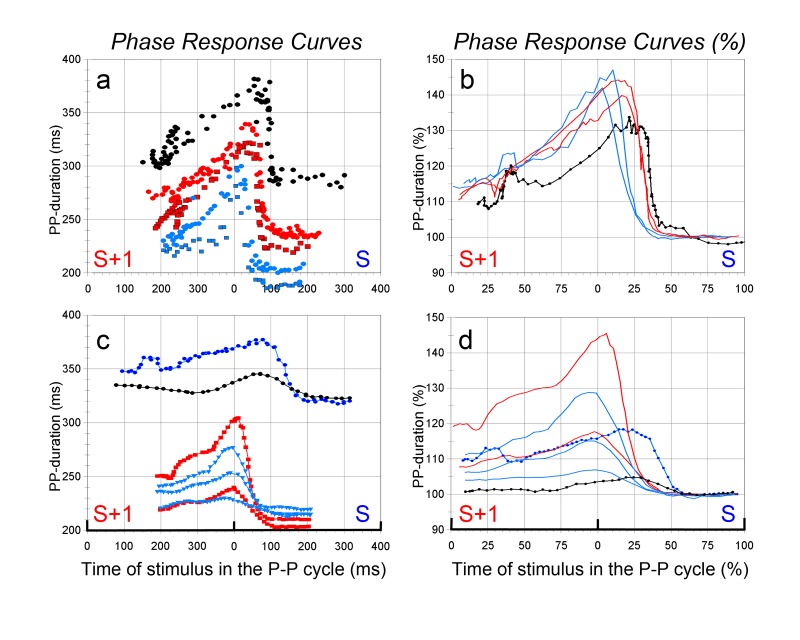
Phase response curves at different baseline P-P cycle durations (layout as in Figure 3). 5a and b are from one animal, baseline manipulated by ß-agonist (light blue symbols) or concurrent stimulation of the other vagus nerve (black symbols). 5c and d: results from 4 different experimental animals. Red and light blue from animals with shorter spontaneous P-P cycles, dark blue and black with longer cycles (due to change in anesthesia from pentobarbital only to the barbiturate-N2O/O2 combination). The various curves have been smoothed by a 5-point averaging filter.

(Figure 2a) At an adjustable delay after a P-wave a burst of 1 to 8 pulses is given, aiming at a prolongation of around 50% of the current cycle length; total duration of the pulse train not exceeding 50 ms (Figures 3 and 5; Figure 4 shows as demonstration the response to a higher stimulus strength). After each burst a rest period is programmed, enough for heart rate to return to control value (as checked from the paper recording) and to (manually) change the delay to scan the whole cycle.

### 2.2. Beat-to-beat (‘pulsatile’) stimulation

**Figure 6.**
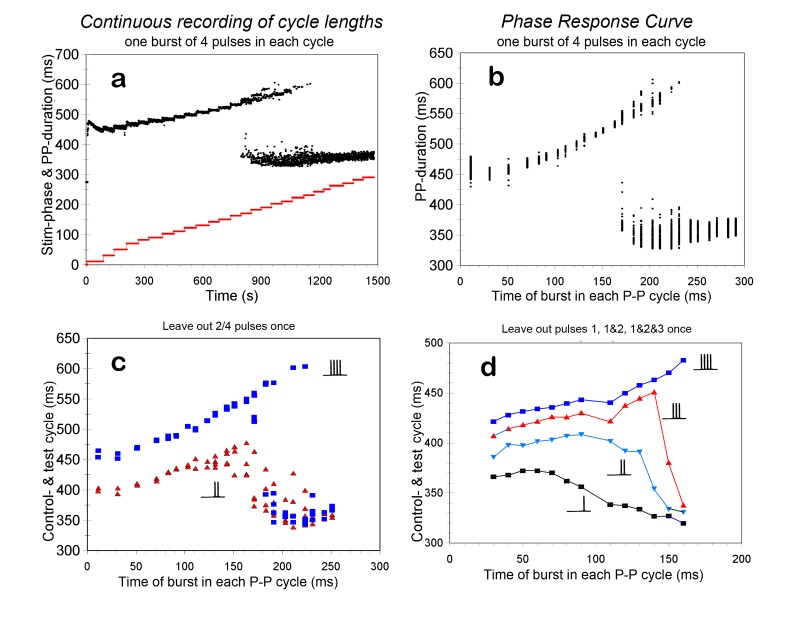
Responses to one burst of 4 pulses in each cycle (15 ms interval, 0.2 ms duration). 6a: Overview of such an experiment; lower (red) curve denotes stepwise increased delay of the stimulus after the P-wave. Upper (black) curve shows successive P-P cycles; each dot represents one cycle. At a delay setting of 170 ms, around 800 secs in the experiment, the cycle durations become more and more unstable at progressively longer delays. 6b: Resulting cycle durations as phase response curve; delays after the P-wave on the abscissa. 6c/d: Phase response curves to decreases of only one stimulus burst in beat-to-beat stimulation. Layout as in 6b. 6c is from the same experiment as in fig. a/b. Blue squares give the duration of the pre-test P-P cycle. In the test cycle the burst is halved to only 2 pulses, red triangles give the resulting duration of the test cycle. 6d is from a different experiment, same burst stimulation settings; from the burst of 4 pulses progressively more are suppressed in the test P-P cycle, as indicated. More and more cycle shortening is observed. (Note: adapted scales in 6d).

**Figure 7.**
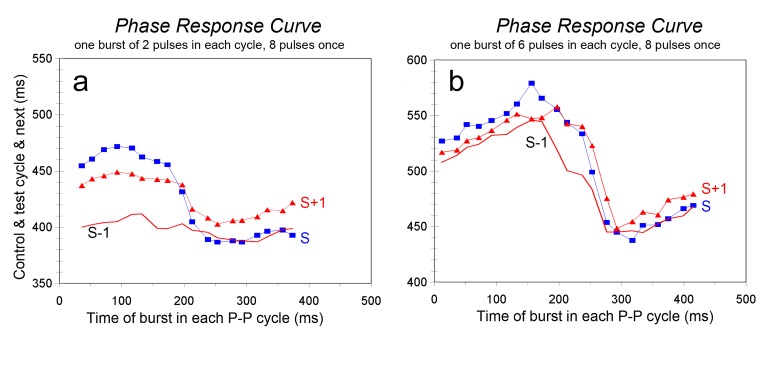
Responses to increases of only one stimulus burst in beat-to-beat stimulation. Drawn red lines give the undisturbed interval durations on beat-to-beat stimulation. 7a: bursts of 2 stimuli, in the test interval increased to 8. Squares: duration of test interval, triangles: next interval. 7b: bursts of 6 stimuli, also increased to 8. Resting heart period around 330 ms.

(Figure 2b1) Each P-wave starts a chosen delay for a burst of pulses as for single burst stimulation. Once a steady state is reached for that delay setting, in one test cycle a programmable number of pulses from the burst is suppressed (Figure 2b2) or extra pulses are added (Figure 2b3). The effect on that cycle (“S”) and the following ones (S+1, …) is plotted. Then the delay-setting is changed and the process is repeated until the whole cardiac cycle has been scanned with different delay settings (Figures 6, 7 and 9).

### 2.3. Continuous (‘tetanic’) stimulation

**Figure 8.**
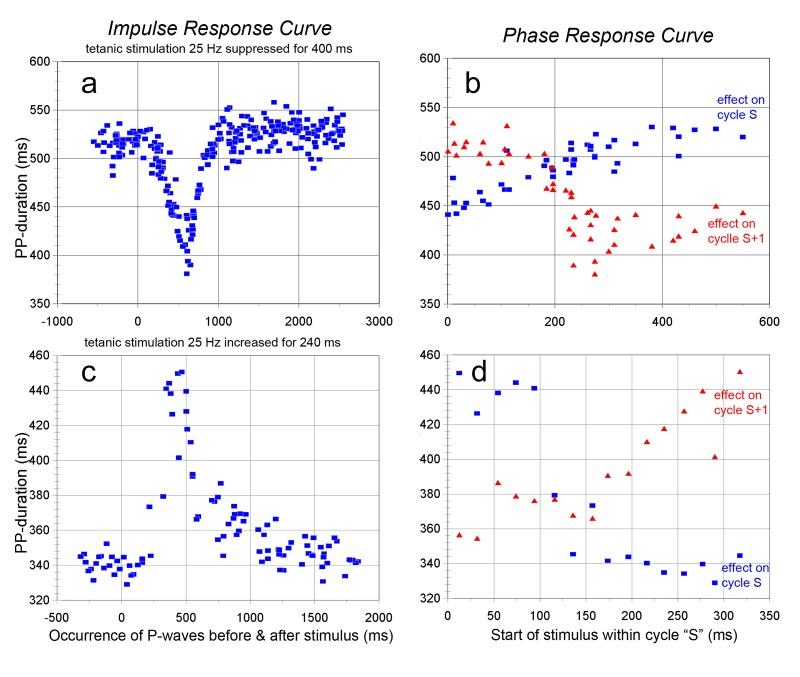
Responses to short lasting decreases or increases of tetanic stimulation at 25 Hz (layout as in Figure 2a/b). 8a/b: suppression of stimulation for 400 ms. 8c/d: frequency increased to 100 Hz for 240 ms. The dots in 8c/d are averages of 2 runs: one with increasing and one with decreasing ‘stimulus’ delays.

(Figures 2c1 and 2c2) A constant stimulation frequency of around 25 Hz is switched on. When a steady-state P-P cycle length is reached, in a given cycle either the stimulation is switched off for a programmable period of time (Figure 2c1) or the frequency is increased for a short period (Figure 2c2), while shifting that event through the cardiac cycle as in protocol 2.1 (Figure 8 and, partly, in Figure 5a, black symbols).

In the instance of fig.5a tetanic stimulation of one vagus nerve served to manipulate P-P cycle length, while the PRC’s were measured in response to single burst stimulation of the other vagus nerve as in protocol 2.1. Alternatively, the P-P cycle was shortened by single i-v dose of the ß-agonist isoproterenol (100 µg Aleudrine, Boehringer, Ingelheim).

## 3. Results

### 3.1. Single short burst stimulation

Figure 3a shows a (computer generated) ‘strip chart recording’ of the cycle lengths at the start of a stimulation series where a burst to the right vagus nerve was scanned through the whole cycle. In the first place the recording demonstrates that a small respiratory sinus arrhythmia was still present, in spite of the bilateral vagotomy. This point is discussed below, in section 3.4. The inserted numbers at the cycle responses indicate the delay time settings in ms. They show that going from 11, 21, 31 to 51 ms in the cycle the effects on the first beat (“S” in Figure 2a) are increasing; at 71 ms the effect already decreases and at the long delays of 151 and 171 ms there is only an effect on the next beat (“S+1” Figure 2a), nothing in the ongoing, stimulated cycle. Figure 3b shows this differently: the delay of the stimulus in the cycle of the sinoatrial node is on the X-axis. The induced cycle duration is on the Y-axis: the blue squares for the first (stimulated) cycle, and the red triangles for the following cycle. This type of curve will be referred to as Phase Response Curve or PRC, although it, strictly speaking, in Figure 3b is the delay from the start of the cycle and not the phase in the P-P cycle on the abscissa (delay divided by duration of the undisturbed cycle as in Figure 3d). The figure shows that not all effect is lost when the stimulus comes too late to prolong the ongoing (first) cycle: there is still an effect on the next one. Indeed, the curves for cycle “S” and cycle “S+1” are, more or less, continuous when placed next to each other, as demonstrated in Figure 3d.

Figure 3c gives the classical representation of results for this series of stimulations. The end of each cycle after the stimulus (which is placed for each stimulation as time = 0) gives its point on the abscissa, the duration of that particular cycle gives the ordinate. The latter may also be expressed in milliseconds prolongation or as percentage of the duration of the last pre-stimulus cycle. This way of plotting will be referred to as 'the impulse response'. Indeed, it gives the impression of an impulse response of some identifiable system; however, it is composed of many, but all different, responses to the same stimulus. Figure 3a also demonstrates that there is still a, be it small, effect detectable after the first two beats. Heart rate returned to baseline values only after 12 seconds, when the next stimulus was applied.

In the following test in the same animal the other (left) vagus nerve was stimulated at a higher intensity. This produced an almost ‘all or none’ response, as shown in Figures 4a-c: although I tried to get an intermediate response, as shown by the density of data points around the transition phase in Figure 4b this did not happen. Interestingly, many of the applied stimuli provoked a shortening of cycle duration in cycles S+1 and/or S+2 before returning to normal, a phenomenon that did not occur in the more moderate stimulation series as in Figure 3.

Variability of the PRC

In the experiment of Figures 3 and 4 the basic cycle length was changed by 2 interventions: it was increased by concurrent tetanic stimulation of the other vagus nerve or decreased by a single dose of isoprenaline (ß-agonist) just before the stimulation series. The resulting PRC’s are shown in Figures 5a and b. Both give the PRC’s over the stimulated and unstimulated cycle, 5a in absolute numbers, 5b (after curve smoothing by 5 point averaging) as percentage of the control cycle, just before the stimulation. The colors in 5a and b describe the same stimulation series: blue the shortened P-P cycle (190-200 ms), red the baseline cycle (230-240 ms), and black the prolonged cycle (290 ms). Figure 5b shows that the longer cycles also tend to have longer ‘sensitive periods’ to vagal stimulation as percentage of the cycle duration. This issue is underlined by the composition of PRC’s from 4 other experimental animals shown in Figures 5c and d. The baseline cycle lengths were different, due to biological variation and factors like applied anesthesia (pentobarbital as sole anesthetic resulted in high resting heart rates, the combination with NO_2_/O_2_ gave lower baseline heart rates). The same line colors have been used for series in one and the same animal. The longer resting cycles come with definitely longer sensitive periods to vagal stimulation as percentage of the cycle duration (Figure 5d).

### 3.2. Beat-to-beat stimulation

In these experiments considerably lower stimulus intensities for the stimulus burst in each cardiac cycle had to be applied than in the previous protocols. Only stimulus bursts could be used that induced weak responses when applied once. Stimulus intensities that, by themselves, would induce a strong response resulted in extremely low heart rates when applied repeatedly, making blood pressure drop to values that would have made further testing impossible. When a suitable combination of pulse intensity, within-burst frequency and burst-duration was found, the cycle was scanned for a number of P-to-stimulus delay-settings, each maintained for at least half a minute.

Two outcomes were found: one, where the shifting of the stimulus burst through the P-P cycle had little effect on the induced new cycle duration: it was prolonged with respect to the resting cycle length, but the amount of prolongation was almost independent of the timing of the vagal burst in the cycle. The alternative outcome, at slightly higher stimulus intensities, was one as shown in Figures 6a and b, presented as time curve and as phase-response curve, respectively.

**Figure 9.**
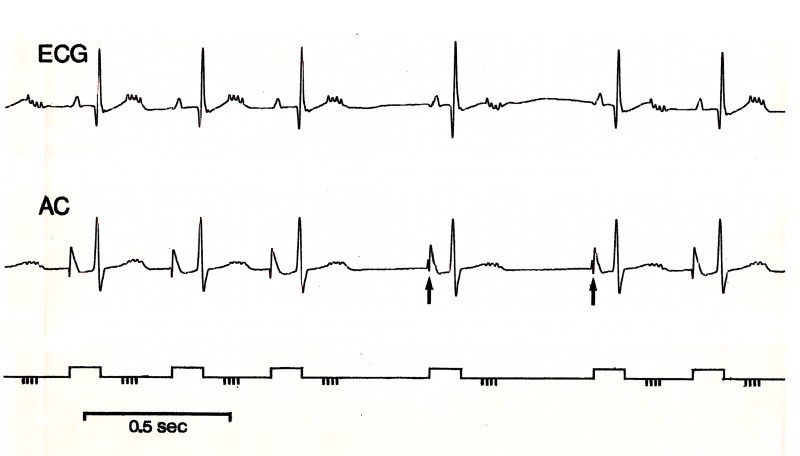
Stretch of the recording from the experiment with beat-to-beat stimulation in fig. 6, delay-setting of the stimulus in the cycle ~ 170 ms. Top: surface ECG, AC: atrial complex from catheter electrode, below: trigger pulse started by atrial depolarization, schematic indication of 4 stimuli. The stimulus artefacts are clearly visible in ECG and AC. The cycle duration jumps between ~ 360 and 550 ms. The arrows point to slightly changed AC’s ending the longer cycles, possibly signs of pacemaker shifts.

After a stabilization period there is an almost linear increase of P-P cycle with increasing burst delay (Figure 6a). However, the obtained extra cycle prolongation is slightly less than the time shift of the stimulus. At a P-stimulus setting of 170 ms the cycle length becomes unstable: some cycles follow the original track and prolong further, for some the stimulation comes too late and they are shorter (Figure 6a/b). This becomes more and more evident at longer P-stimulus delays, until at 230 ms all cycles fall in the latter category, be it at an increased standard deviation compared to before the unstable region. There proved to be a definite periodicity in the jumping up and down of the P-P cycle, i.e. it followed the period of the ventilator (just below 2 seconds: 31 breaths/min). Both the atrial catheter and the surface ECG showed subtle signs of changes in atrial activation in synchrony with the P-P cycle periodicity, which might point to shifts of the pacemaking region within the sinoatrial node (Figure 9). This issue is elaborated in section 3.4.

#### 3.2.1. Beat-to-beat stimulation- with suddenly decreased stimulus strength

In the experiment of Figure 6a/b the stimulus in each beat consisted of 4 pulses to the right vagus nerve. In another run in the same experimental animal, using the same settings, the stimulus was decreased by suppressing the first two pulses of just one such burst. In Figure 6c the resulting cycle length is compared to the last ‘normal’ cycle with 4 pulses, just preceding the test cycle. For the stimuli early in the cycle there is an immediate shortening of the ongoing cycle by about 50 ms. However, the later the burst occurs in the cycle, the more unstable this result becomes and especially in the region where the cycle lengths become unstable (170 ms and up) the larger the shortening of the test cycle becomes. The test cycle duration can now be found at the lower ‘twig’ of the bifurcation in Figure 6b. Finally, there is no longer a shortening effect by the decreased burst on the ongoing beat, but the next cycle is shortened by 35 ms. At the short P-stimulus delays the one after the test cycle is already back to control values; data not shown. The same pattern was found in other experiments, where the stimulus strength was changed from 4 pulses down to 1 pulse in the test beat. An example is shown in Figure 6d.

#### 3.2.2. Beat-to-beat stimulation with suddenly increased stimulus strength

This experiment is, as it were, the mirror of the previous one, and the results can very well be interpreted as such. Figure 7a shows the case where 2 pulses/cycle had a slight prolonging effect on the P-P cycle, almost independent of the timing in the cycle. Addition of 6 pulses once to such a burst exposed the underlying PRC: early prolonged bursts would increase the P-P cycle from around 400 to 460 ms; late extra pulses had no effect on the ongoing cycle and only a minor effect on the next one. In the alternate case, shown in Figure 7b the bursts were increased from 6 to 8 pulses in the test cycle. The PRC was already apparent in the ‘background’ effect induced by 6 pulses/cycle; addition of 2 extra pulses worked only for the early bursts in the ongoing cycle, with some remnant effect on the next cycle for late bursts. However, in the transition phase, where the effect of 6 pulses was unstable, the addition of 2 extra pulses had a large effect on the ongoing cycle and the next one.

### 3.3. Continuous (‘tetanic’) stimulation

This type of vagus nerve stimulation has been studied extensively in the literature. Rather unexpectedly I found very variable heart rates from one beat to the next, even though the stimulation frequency was held constant. In the present experiments only the effects of short-lasting increased or decreased frequency blocks was studied. Compared to the previous - pulsatile - modes of stimulation it was striking how much more/longer the test blocks had to be to provoke heart rate effects that would clearly stand out of the background variability. The issue of ‘vagal noise’ is elaborated below in section 3.4.

#### 3.3.1. Tetanic stimulation with short suppression of stimuli

Figure 8a/b shows an example of such an experiment: tetanic stimulation at 25 Hz had increased cycle length from a resting 290 ms to around 520 ms. Suppression of stimulation for 400 ms led to a maximal shortening effect to around 400 ms (Figure 8a), obtained in the second cycle after the suppression of stimuli had started (Figure 8b). The PRC for cycles 1 and 2 demonstrate that only a small effect was reached in the first cycle, if stimuli had been stopped in the first 200-250 ms; moreover, that shortening effect was already gone in the second cycle. The largest effects occurred in the second cycle for those instances where the full block of stimuli had occurred just before the end of the first which it had left uninfluenced. The P-P cycle was quickly back to earlier values, even slightly overshooting those for a short period of time.

#### 3.3.2. Tetanic stimulation with short-lasting frequency increase

This experiment should come out more or less the same as the one described in Figure 5a/b (black symbols), where on one vagus nerve the tetanic frequency was applied, while the other nerve served to apply a short-lasting extra stimulus. Here one and the same nerve and same stimulus intensity settings were used to, shortly, increase the frequency. This encountered the same problems as mentioned under 3.3.1: a sizeable change had to be applied to induce a response that would clearly stand out of the background noise. Since this protocol also implied intense use of the same vagus fibers over and over, fading of the response was observed in the course of a test run [20]. Therefore, the result in Figure 8c/d is an average of 2 such runs: one where the delay setting was going up and the other where it was going down (from 10 to 320 ms and reverse). The baseline cycle of 260 ms was prolonged by the tetanic stimulation to 340 ms. Both the impulse response curve and the PRC look like the ones shown in section 3.1, with one exception: depending on the circumstances the apparent delay of the impulse may turn out much longer than the value of around 100-120 ms that can be read from Figure 3, rather more like 200 ms.

### 3.4. Unsolicited results

In the present experiments, where both vagi have been cut, one would expect a stable baseline of heart rate, devoid of the fast beat-to-beat variations, if these were only vagally mediated. Rabbits are known to make an exception to that rule: even after bilateral vagotomy a small respiratory sinus arrhythmia (RSA) of a few milliseconds peak-to-peak can still be observed [21]. In all result curves shown above this caused some extra background variability, above and beyond the biological variation that occurs anyway.

The situation is, however, more complicated: depending on the applied stimulus mode, the observed RSA was amplified or dampened. In the beat-to-beat stimulation (protocol 2) it, generally, was amplified, extremely so when the stimulus was applied in the ‘unstable’ region (Figure 6 and Figure 9, below); under tetanic stimulation (protocol 3) it was not always observed, or it sort-of drowned in a high-frequency background noise of fast beat-to-beat variability without a defined predilection frequency, as confirmed by frequency analysis of the time series (results not shown).

## 4. Discussion

The main objective of this study was to investigate how the physiological properties of the vagus nerve-sinoatrial node complex in vivo translate changes in cardiac vagal activity into heart rate variability. To do this, electrical stimulation of the efferent vagus nerves was applied after bilateral vagotomy. As shown in the results section, the heart rate effects of (changes in) vagal activity depend strongly on the timing of this activity in the sinoatrial node cycle and on the prevailing conditions.

An overwhelming amount of literature has laid a solid basis for understanding of how the parasympathetic system influences the functioning of the cardiovascular system, dating back to 1868 with the work of Donders, who for the first time called attention to the phase dependency of the heart rate effect of vagal stimulation [15,17,22-30]. In many of those studies this effect and those of different stimulation frequencies have been investigated, however, the issue of temporary increase or decrease of stimulus intensity has received very little attention.

It may seem that this issue is covered by modulation of the frequency of vagus nerve stimulation, going up and down following a sinusoidal pattern. When sinusoids of a broad range of frequencies are used, one may assume to have caught all dynamic aspects of the relation between vagus activity and sinoatrial node response (“transfer function analysis”), as it has been applied by a number of authors [31-34]. Of these the experiments by [35] come closest to what has been presented here: these authors applied stimulation coupled to the P-wave, which was slowly frequency modulated, modulation frequency spanning at least several heartbeats. However, that type of system analysis supposes a more or less linear system, not one where the timing of a stimulus may induce different effects, depending on its arrival time in the cardiac cycle. This relates to a basic issue that is haunting the field of HRV: heart beats, or rather R-R-intervals, are what is being measured on a beat-to-beat scale which has its own dynamic, due to processes like the ones studied in this paper. Therefore, newly derived numbers like sample entropy or multiscale entropy are using the original R-R-interval series [36,37]. In some other analysis methods, it has become customary to interpolate the RR-values to have a signal that is regularly sampled at fixed intervals [32,33,35]. This serves to solve the problem that most processes also have a time-dependent component, and representations of HRV where the timescale is not readily apparent, cannot easily be interpreted. This becomes an even more pressing problem if there are substantial changes of the prevailing heart rate in the course of the observation period. Interpolation of the original time series by whichever method [38-40] cannot redress this problem. As is shown in this study, low heart rate will show dynamics different from that at higher heart rates, be it alone by the changed PRC and the timing of vagal bursts in the cycle.

All through this study the Phase Response Curve has been shown to be of paramount importance to understand the effect of a cardiac vagal impulse on heart rate. Also that observation is not new; PRC’s have often been studied, e.g. in [17,30,41], however, the application of repeated, short-lasting stimulus bursts was different from the approach chosen here: those authors used the timing of the bursts as the driving mechanism, independent of the beat occurrences, to find regions of ‘entrainment’ (e.g. [14,16]) where HR would follow that timing. One might think of vagal bursts induced by expiration as a more or less independent mechanism to deliver such a driving signal. However, respiration rate is generally (much) lower than heart rate and lower than the applied rate of vagal burst stimulation in those entrainment experiments. In vivo, roles are reversed: the vagal bursts follow each heartbeat, as they are locked to the pulse wave upstroke by way of the baroreflex.

In Figures 3 and 4 it was demonstrated that the heart rate reaction to a vagal burst has two components: 1) an immediate, strong effect, lasting at most one beat and the next and 2) a slow after-effect that may last for several seconds. The immediate response is phase-sensitive; a burst coming too late in the ongoing cycle may at most have a diminished effect on the next one. The slow after-effect is not as phase-sensitive as that and it is only small, compared to the immediate effect. However, it builds up with repeated stimulation and requires many seconds to disappear. That may be why, in the case of tetanic stimulation, it is so difficult to induce an immediate, sizeable cycle shortening, and even with prolonged suppression of the ongoing stimulation cycle length does not fall back to the unstimulated value (Figure 8a/b).

Beat-to-beat stimulation early in the cycle gave the largest and most stable responses; when the stimulation occurred in the region where the response became unstable, a small disturbance like that caused by respiration (be it artificial or spontaneous ventilation) could impose its rhythm on the unstable situation, consequently inducing a large RSA in the face of an unchanging vagal stimulus to the heart (Figure 6). To my knowledge this has not been described in literature before. When the stimulus came late in the cycle, a more or less stable effect on the following cycle was observed, less effective though than stimulation early in the cycle (but still with a sizable RSA).

The above does not explain the results of tetanic low-frequency stimulation. Since the pulses were not locked to the P-wave, there was variability in where and how many pulses would occur in the sensitive period of the prevailing PRC, later coming pulses in that cycle are ineffective. This process may be expected to lead to some variability in cycle times. Additionally, the transfer in the cardiac ganglia is probably not of the one-to-one type; since the ganglions cells are known to display autonomous subthreshold oscillations and spontaneous activity [42], interference with the imposed stimulation frequency might act as an additional cause of variability. Most of the cycle prolongation under this type of stimulation is probably due to what was called above the ‘built-up after-effect’. Consequently, only when the suppression of stimulation starts very early (and inhibits a sizeable number of pulses) would the first cycle be shortened (Figure 8a/b), the largest effect to occur in the next cycle. The opposite: a short-lasting block of increased frequency behaves just like the impulse responses described in the beginning, now against the background of an increased cycle length (Figure 8c/d).

### 4.1. The baroreflex, hrv and timing of vagal bursts

This study provides more insight into the working of the baroreflex on heart rate. One may now ask, how important is this PRC to the responsiveness of the sinoatrial node to its in vivo vagal input. In other words, in particular when looking at Figure 6, what does this mean for the normally arriving vagal burst after a ventricular contraction? In the experiments stimulation was coupled to the atrial complex, since the heart rate is generated in the atrium, and coupling to the (ventricular) R-wave is inherently biased by possible timing differences due to the vagal (and other -) effects on AV-conduction. Figure 9 shows the surface ECG and atrial complex during repeated stimulus bursts ~170 ms after the P-wave, i.e. in the unstable phase of the sinoatrial node cycle. The AV-conduction is slightly prolonged, the vagal burst occurs around the T-wave, i.e. some 80-100 ms after ventricular contraction has started. This is around the very first moment that a vagal response to baroreceptor afferent activity might have been expected in rabbits, if we assume the estimates of reflex latency in the cat to be applicable to rabbits as well [10,12,43], where values of 60-70 ms, 26-90 ms and 20-60 and/or 70-110 ms respectively were found. The latency of 100-120 ms which I found in my thesis work in awake rabbits from HR responses to carotid sinus nerve and depressor nerve stimulation, is slightly longer [44]. In our experiments in humans [45] we estimated still longer reflex times, extrapolating from electrical stimulation of carotid sinus nerves to heart rate responses (0.35 s). However, in view of a number of uncertainties in those measurements, which have later been stressed by Eckberg and coworkers [46] this estimate might be over-cautious and therefore too long.

Therefore, although these are experiments in rabbits, the results have relevance for baroreflex functioning and related HRV in human physiology. This holds in particular for the regulation at quiet heart rates, below 75 beats/min [47], where changes in systolic pressure in the ongoing beat are known to influence the occurrence of the upcoming P-wave. A more elaborate discussion of this issue has been given in [48] and [45]. In the computation of baroreflex sensitivity (BRS) the original algorithm is to correlate the systolic pressures in the rising phase of a phenylephrine induced pressure rise to the durations of the succeeding beats [49]. At heart rates lower than 75 this should be the duration of the very beat where the systolic pressure was measured. From the experiments presented here one may infer that the heart rate lowering is even more effective if the vagal pulses arrive still earlier in the cycle, therefore at substantially lower heart rates than 75/min. HRV is known to diminish at higher HR, this is probably due to the vagal pulses coming too late for the ongoing beat to have their full effect, so it is delayed –with decrement- to the next beat. This also explains the increasing beat delay observed at higher heart rates in the systolic pressure to heart period correlation such as used in the running baroreflex sensitivity xBRS [50] or the phase delay between systolic pressure and heart period changes in Fourier analysis [1]. This delay need not necessarily be a sign of increasing sympathetic involvement in the baroreflex to heart rate response. Since this effect of diminishing response to the same vagal burst activity does not depend on the frequency of some underlying oscillation, be it respiratory (high frequency or “HF”) or sympathetically mediated 10-seconds oscillation (low frequency or “LF”), one cannot predict from this how a change in HR might affect a quotient like LF/HF [51]. Other typically vagal measures, like RSA or BRS, will definitely give lower numbers.

### 4.2. Respiratory sinus arrhythmia and the vagus nerve

As was shown above, even after bilateral vagotomy RSA was observed. In the literature this phenomenon has been attributed to direct stretch of the sinoatrial node [21,52]. It might equally well be due to cardiac stretch receptors synapsing with efferent autonomic nerves in the ganglionic plexus near the heart [7,53]. In the present experiments RSA increased dramatically when the vagus was stimulated at a critical timing in the cycle of the sinus node (Figures 6 and 7). Whatever the exact cause of RSA in the resting situation, from physics we know that a minute disturbance may tip the balance in an unstable system; respiratory movement might be just that disturbance. This might also be an explanation for the extreme RSA that is sometimes observed in young persons, where HR may jump from around 60 to 100 bpm and back in a few beats (personal observation).

### 4.3. The sinus node under vagal control

**Figure 10.**
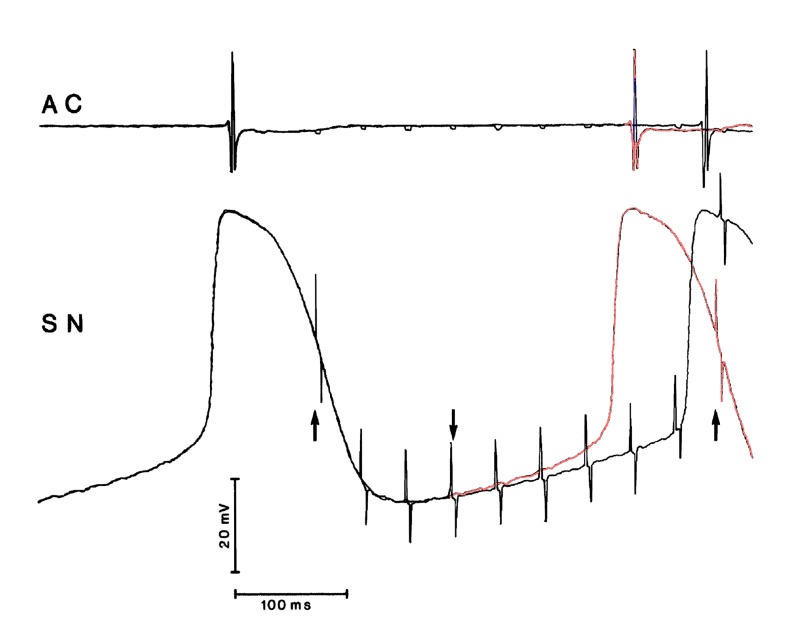
Recording from an experiment by L.N. Bouman and F.I.M. Bonke: micro-electrode in rabbit sinoatrial node cell (SN) while the attached vagus nerves were stimulated. AC: atrial complex, recorded from the crista terminalis. At the rightmost up-arrow 25 Hz vagus nerve stimulation started. The last unstimulated and first stimulated cycles have been superimposed (repeated up-arrow to the left). Stimulation is evident from the large artefacts. At the down-arrow the first change from unstimulated to stimulated action potential is detectable. The earlier this occurs in the cycle, the larger the effect on the ongoing depolarization. For clarity the last unstimulated cycle has been touched up in red from that point onward. Figure has been published earlier in [44] as Figure 3.2.

All along this paper has been about the originator of heart rate, the sinus node, and how it reacts to vagal influences. A number of basic issues related to known vagus nerve and sino-atrial node function should still be discussed. First of all, the delay time between arrival of acetylcholine at the end organ and the first measurable change of function in that organ. It is well-known that this delay after activation of the (postganglionic) muscarinic receptor is much longer than that at the nicotinic receptor (like the ones in the ganglia and the neuromuscular junction). The latter one has delay times in the order of 0.2 ms, the former in the order of 100-150 ms [54,55]. This implies that the shortest latency between vagus nerve stimulation and shifting the next P-wave to a later moment is very close to this ‘muscarinic transmission delay’ in the present experiments (Figure 3). Second, which phase of the sinoatrial node action potential should be ‘hit’ by vagal pulses to obtain most effect? I was given the opportunity by Drs. L.N. Bouman and F.I.M. Bonke to re-analyze an archived copy of their experiments on vagus nerve stimulation in isolated rabbit sino-atrial node preparations [56]. They measured the sinoatrial node response to stimulation by trains of 25 Hz lasting a few seconds. The responses in these rabbits were unlike those in Jalife and Moe’s experiments in a comparable preparation in young cats [57]. The latter authors applied strong stimulus bursts, to which the sinoatrial node would react by a full ‘reset’, i.e. the action potential was pushed from its depolarization course back to the maximal hyperpolarization level and had to restart from the lowest point (Figure 1 more or less depicts this situation). The starts of a tetanic stimulation by Bouman and Bonke were like the one demonstrated in Figure 10, where no reset but a gradual deviation from the unstimulated course would show.

Comparing the occurrence of the atrial complex in Figure 10 to the PRC’s observed in my own experiments (started by the atrial depolarization as well) the most effective timing of a single stimulation burst is in the late repolarization-early diastolic phase of the sinus node action potential. This is in good agreement with Jalife and Moe’s [57] measurements.

Finally, the short- vs. the long-lasting effects of vagal stimulation; is there a basis in known sinoatrial node properties? An extensive review of the literature has been published in [58] ranging from the various types of muscarinic receptors in sinoatrial node tissue to the membrane channels involved in the translation of acetylcholine effect to changed membrane channel properties. It may be concluded that the hypothesis formulated above has a sufficiently sound basis in the known properties of the sinoatrial node, i.e. vagal influences may be distinguished in fast and short-lasting, explaining the sudden prolongations of cycle length, vs. slowly building up effects of repeated stimulation and the relatively slow return to the baseline values afterwards. Only the fast influence on HR of a vagal burst can be quickly undone from one heartbeat to the next, the slow response takes several beats to disappear.

### 4.4. HRV and the vagus nerve

The cardiac vagus nerve fibers, serving as efferent pathway to the baroreflex, provide immediate adaptation of heart rate to changes in blood pressure. Therefore, the efferent cardiac vagal bursts, one per heartbeat, might be considered translations of the incoming baroreceptor afferent bursts that are caused by pulsatile stretch of the vessel wall at each heartbeat. However, this conversion is not one-to-one: already in the first transmission station, the nucleus tractus solitarii of the medulla oblongata, integration takes place with other incoming visceral and somatic afference [10,43]. In addition, the central interaction with respiration induces a partial blockade of vagal outflow during the inspiratory phase [10,43,59]. Consequently, the outgoing cardiac vagal traffic is not only a reflex response to the baroreceptive input signals. Moreover, the ganglionic cardiac plexus is not the simple textbook transmission station; ganglion cells themselves show subthreshold oscillations and, on top of that, incoming neural traffic from cardiac sensory neurons may alter their activity [7].

The combined effects of heart rate on the effectiveness of incoming vagal traffic, i.e. the change in duration of the ‘sensitive period (shorter at higher HR) and the relatively late arrival of vagal bursts are good explanations for the observed relationship between HRV and HR, mentioned in the introduction [4,5]. This does not settle the ‘hot’ issue whether it is better to take heart period or heart rate for the calculations of HRV. Parker et al. showed in 1984 [60] a nice linear correlation between amount of vagal activity (stimulus pulses) and obtained heart period, an observation that underlined Jewett’s earlier finding that the number of preceding cardiac vagal impulses correlated well with the length of the heart period [11]. These observations have given credence to the corollary that heart periods should best be measured to account for vagal effects (mainly in the resting condition) and heart rates during exercise, looking at the effect of the sympathetics. When looking at the 1/x relationship between the two, one may also argue that in the resting condition heart period changes would almost vanish if expressed as HR-changes, and vice versa. A more elaborate discussion of the issue has been given earlier [61].

The issue raised by Yaniv et al. [6,62] relating to inherent stochastic behavior of the sinoatrial node as cardiac pacemaker is yet another matter. Pacemaker cells, when isolated in tissue culture, display widely varying intervals between firings [63]. Once the cells have replicated and form a syncytial-like tissue, the interval becomes stabilized. In vivo it is a known fact that cells in the pacemaking region may show different behaviors: from ‘true pacemaking cells’ where the earliest activity in the cycle can be recorded, to ‘latent pacemaking cells’ which display a slower diastolic depolarization and a sharp transition to the systolic upstroke [64,65]. The postganglionic sympathetic and vagal fibers have an uneven distribution over that area, consequently the actual pacemaking region may shift with autonomic activity [56]. An example of that may be observed in Figure 9. Even more extreme might the sinoatrial node be blocked to such an extent, that a vagal escape occurs and the actual pacemaker shifts to another pacemaking area, like the AV-node, for one or more beats. That did not occur in the present experiments, where care was taken not to go to extremes with vagal stimulation, but the phenomenon of vagal escape is well-known in physiological literature [66,67] and, of course, from clinical observations like in ophthalmic surgery [68] where manipulation of the eye can produce strong sinoatrial node inhibition, and the AV-node temporarily may take over pacemaking of the heart.

### 5. Conclusions

In this series of experiments, the importance of the Phase-Response Curve for understanding vagally induced phasic heart rate changes has been established, both for single burst and for beat-to-beat burst-like stimulation. The latter can be considered the normal in-vivo mode of operation in response to baroreceptor input due to the pulse wave upstroke, making vagal activity the most important contributor to HRV in the healthy, supine resting condition. The experiments have also shown that the PRC is not a constant given, but a changing operator, depending on the prevailing condition of the sinoatrial node and immediately prior vagus nerve activity. Under conditions the response to a vagal burst may become unpredictable, when its timing in the cycle has become critical. If vagus nerve activity does not come beat-to-beat, but as a more or less continuous background ‘vagal tone’, it induces slower on- and off-responses than those provoked by burst-like activity.

In general, the HR-response to vagal activity shows two components: a fast one that may provoke large cycle changes from one beat to the next, and a slow one, that builds up with longer lasting activity. The latter one also takes longer to subside, in small steps.

The response to vagal stimulation is not only determined by the properties of primary pacemaking cells in the sinoatrial node, but also by the properties of the ganglion cells in the cardiac plexus and by the unequal distribution of their end-organ effects, leading to pacemaker shifts within the sino-atrial node or even to other areas, like the AV-node. The existence of RSA, even after vagotomy, may be explained by integrating action of the cardiac autonomic plexus where vagal, sympathetic and cardio-sensory information come together.

### 6. Limitations

Blood pressure decrease during vagal stimulation may have induced increased sympathetic drive to the sinoatrial node (However, control experiments after ß-blockade by propranolol did not give remarkably different results). The change in applied anesthesia in the course of the experimental series, from barbiturate-only to barbiturate and N2O/O2, in order to have animals with lower baseline heart rates, may have induced more variable heart rates at the same time.

## References

[1] DeBoer RW, Karemaker JM, Strackee J (1987). Hemodynamic fluctuations and baroreflex sensitivity in humans: a beat-to-beat model. Am J Physiol.

[2] Karemaker JM, Wesseling KH (2008). Variability in cardiovascular control: the baroreflex reconsidered. Cardiovascular Engineering.

[3] Karemaker JM (2009). Counterpoint: Respiratory sinus arrhythmia is due to the baroreflex mechanism. Journal of applied physiology.

[4] Billman GE (2013). The effect of heart rate on the heart rate variability response to autonomic interventions. Frontiers in Physiology.

[5] Monfredi O, Lyashkov AE, Johnsen A-B, Inada S, Schneider H, Wang R, Nirmalan M, Wisloff U, Maltsev VA, Lakatta EG, Zhang H, Boyett MR (2014). Biophysical characterization of the underappreciated and important relationship between heart rate variability and heart rate. Hypertension.

[6] Yaniv Y, Tsutsui K, Lakatta E (2015). Potential effects of intrinsic heart pacemaker cell mechanisms on dysrhythmic cardiac action potential firing. Frontiers in Physiology.

[7] Armour JA (2008). Potential clinical relevance of the ‘little brain' on the mammalian heart. Experimental Physiology.

[8] Karemaker J (1995). Chaos in heart rate and the vagus nerve. A theoretical and experimental investigation.

[9] Karemaker JM (2014). Vagal effects on heart rate: Different between up and down. Cardiovascular Oscillations (ESGCO).

[10] Iriuchijima J, Kumada M (1964). Activity of single vagal fibers efferent to the heart. The Japanese Journal of Physiology.

[11] Jewett DL (1964). Activity of single efferent fibers in the cervical vagus nerve of the dog, with special reference to possible cardio-inhibitory fibres. The Journal of Physiology.

[12] Kunze DL (1972). Reflex discharge patterns of cardiac vagal efferent fibres. J Physiol (London).

[13] Levy MN, Martin PJ (1981). Neural regulation of the heartbeat. Ann Rev Physiol.

[14] Guevara MR, Glass L (1982). Phase locking, period doubling bifurcations and chaos in a mathematical model of a periodically driven oscillator: A theory for the entrainment of biological oscillators and the generation of cardiac dysrhythmias. J Math Biol.

[15] Levy MN, Martin PJ, Iano T, Zieske H (1969). Paradoxical effect of vagus nerve stimulation on heart rate in dogs. Circ Res.

[16] Michaels DC, Slenter V. A. J., Salata JJ, Jalife J (1983). A model of dynamic vagus-sinoatrial node interactions. Am J Physiol.

[17] Abramovich-Sivan S, Akselrod S (1998). A phase response curve based model: effect of vagal and sympathetic stimulation and interaction on a pacemaker cell. J Theo Biol.

[18] UFAW Staff (1967). The UFAW handbook on the care and management of laboratory animals.

[19] Karemaker JM, Borst C, Schreurs AW (1980). Implantable stimulating electrode for baroreceptor afferent nerves in rabbits. Am J Physiol.

[20] Salata JJ, Jalife J (1985). "Fade" of hyperpolarizing responses to vagal stimulation at the sinoatrial and atrioventricular nodes of the rabbit heart. Circ Res.

[21] Perlini S, Solda PL, Piepoli M, Sala-Gallini G, Calciati A, Finardi G, Bernardi L (1995). Determinants of respiratory sinus arrhythmia in the vagotomized rabbit. Am J Physiol.

[22] Donders FC (1868). Zur Physiologie des Nervus Vagus. Pflügers Archiv ges Physiologie.

[23] Brown GL, Eccles JC (1934). The action of a single volley on the rhythm of the heart. J Physiol (London).

[24] Brown GL, Eccles JC (1934). Further experiments on vagal inhibition of the heartbeat. J Physiol (London).

[25] Levy MN, Martin PJ, Iano T, Zieske H (1970). Effects of single vagal stimuli on heart rate and atrioventricular conduction. Am J Physiol.

[26] Stuesse SL, Levy MN, Zieske H (1978). Phase-related sensitivity of the sinoatrial node to vagal stimuli in the isolated rat atrium. Circ Res.

[27] Levy MN, Wexberg S, Eckel C, Zieske H (1978). The effect of changing interpulse intervals on the negative chronotropic response to repetitive bursts of vagal stimuli in the dog. Circ Res.

[28] Dong E, Reitz BA (1970). Effect of timing of vagal stimulation on heart rate in the dog. Circ Res.

[29] Levy MN, Lano T, Zieske H (1972). Effects of repetitive bursts of vagal activity on heart rate. Circ Res.

[30] Jalife J, Slenter VA, Salata JJ, Michaels DC (1983). Dynamic vagal control of pacemaker activity in the mammalian sinoatrial node. Circ Res.

[31] Peňáz J (1962). Frequency response of the cardiac chronotropic action of the vagus in the rabbit. Arch Physiol Biochem.

[32] Berger RD, Saul JP, Cohen RJ (1989). Transfer function analysis of autonomic regulation. I. Canine atrial rate response. Am J Physiol.

[33] Saul JP, Berger RD, Chen MH, Cohen RJ (1989). Transfer function analysis of autonomic regulation. II. Respiratory sinus arrhythmia. Am J Physiol.

[34] Saul JP (1996). Transfer function analysis of cardiorespiratory variability to assess autonomic regulation. Clin Sci (Colch).

[35] Mokrane A, LeBlanc AR, Nadeau R (1995). Transfer function analysis of vagal control of heart rate during synchronized vagal stimulation. Am J Physiol.

[36] Richman JS, Moorman JR (2000). Physiological time-series analysis using approximate entropy and sample entropy. Am J Physiol Heart Circ Physiol.

[37] Costa M, Goldberger AL, Peng CK (2002). Multiscale Entropy Analysis of Complex Physiologic Time Series. Phys Rev Lett.

[38] de Boer RW, Karemaker JM, Strackee J (1985). Description of heart-rate variability data in accordance with a physiological model for the genesis of heartbeats. Psychophysiology.

[39] Berger RD, Akselrod S, Gordon D, Cohen RJ (1986). An efficient algorithm for spectral analysis of heart rate variability. IEEE Trans Biomed Eng.

[40] Camm AJ, Malik M, Bigger JT, Breithardt G, Cerutti S, Cohen RJ (1996). Heart Rate Variability - Standards of Measurement, Physiological Interpretation, and Clinical Use [Review]. Circulation.

[41] Slenter VA, Salata JJ, Jalife J (1984). Vagal control of pacemaker periodicity and intranodal conduction in the rabbit sinoatrial node. Circ Res.

[42] McAllen RM, Salo LM, Paton JFR, Pickering AE (2011). Processing of central and reflex vagal drives by rat cardiac ganglion neurones: an intracellular analysis. J Physiol.

[43] McAllen RM, Spyer KM (1978). The baroreceptor input to cardiac vagal motoneurones. J Physiol.

[44] Karemaker JM (1980). Vagal effects of the baroreflex on heart rate [PhD thesis].

[45] Borst C, Karemaker JM (1983). Time delays in the human baroreceptor reflex. J Auton Nerv Syst.

[46] Seidel H, Herzel H, Eckberg DL (1997). Phase dependencies of the human baroreceptor reflex. Am J Physiol Heart Circ Physiol..

[47] Pickering TG, Davies J (1973). Estimation of the conduction time of the baroreceptor-cardiac reflex in man. Cardiovasc Res.

[48] Karemaker JM, Borst C, Sleight P (1980). Measurement of baroreflex sensitivity in hypertension research. Arterial baroreceptors and hypertension.

[49] Smyth HS, Sleight P, Pickering GW (1969). Reflex regulation of arterial pressure during sleep in man. A quantitative method of assessing baroreflex sensitivity. Circ Res.

[50] Westerhof BE, Gisolf J, Stok WJ, Wesseling KH, Karemaker JM (2004). Time-domain cross-correlation baroreflex sensitivity: performance on the EUROBAVAR data set. J Hypertens.

[51] Force T (1996). Heart rate variability. Standards of measurement, physiological interpretation, and clinical use. Task force of the European society of cardiology and the North American society of pacing and electrophysiology. Eur Heart J.

[52] Golenhofen K, Lippross H (1969). Mechanishce Koppelungswirkungen der Atmung auf den Herzschlag [Mechanical coupling effects between respiration and heart rhythm]. [German]. Pflügers Arch.

[53] Bolter CPWSJ (1999). Influence of right atrial pressure on the cardiac pacemaker response to vagal stimulation. Am J Physiol Regul Integr Comp Physiol.

[54] Purves RD (1974). Muscarinic excitation: a microelectrophoretic study on cultured smooth muscle cells. Br J Pharmacol.

[55] Purves RD (1976). Function of muscarinic and nicotinic acetylcholine receptors. Nature.

[56] Bouman LN, Gerlings ED, Biersteker PA, Bonke FIM (1968). Pacemaker shift in the sino-atrial node during vagal stimulation. Pfluegers Arch.

[57] Jalife J, Moe GK (1979). Phasic effects of vagal stimulation on pacemaker activity of the isolated sinus node of the young cat. Circ Res.

[58] Demir SS, Clark JW, Giles WR (1999). Parasympathetic modulation of sinoatrial node pacemaker activity in rabbit heart: a unifying model. Am J Physiol Heart Circ Physiol.

[59] Gilbey MP, Jordan D, Richter DW, Spyer KM (1984). Synaptic mechanisms involved in the inspiratory modulation of vagal cardio-inhibitory neurones in the cat. J Physiol.

[60] Parker P, Celler BG, Potter EK, McCloskey DI (1984). Vagal stimulation and cardiac slowing. J Auton Nerv Syst.

[61] Faes TJ, De Neeling NN, Kingma R, TenVoorde BJ, Karemaker JM (1995). On the quantification of heart rate changes in autonomic function tests: relations between measures in beats per minute, seconds and dimensionless ratios. Clin Sci (Colch).

[62] Yaniv Y, Lyashkov AE, Lakatta EG (2013). Impaired signaling intrinsic to sinoatrial node pacemaker cells affects heart rate variability during cardiac disease. J Clin Trials.

[63] Jongsma HJ, Tsjernina L, de Bruijne J (1983). The establishment of regular beating in populations of pacemaker heart cells. A study with tissue-cultured rat heart cells. J Mol Cell Cardiol.

[64] Lu H-H, Lange G, McC. Brooks C (1965). Factors Controlling Pacemaker Action in Cells of the Sinoatrial Node. Circ Res.

[65] Bouman LN, Jongsma HJ (1986). Structure and function of the sino-atrial node: a review. Eur Heart J.

[66] Hill L, Barnard H (1897). The Influence of the Force of Gravity on the Circulation Part II. Section I. The action of the respiratory pump. Section II. The escape of the heart from vagal arrest. Section III. The mean pressure of the vascular system. J Physiol.

[67] Wallace AG, Daggett WM (1964). Pacemaker activity during vagal escape rhythms. Circ Res.

[68] Donlon JV (1988). Anesthesia for ophthalmologic surgery. ASA Refresher Courses in Anesthesiology.

